# Use of Prescription Antiemetics Among US Commercially Insured Pregnant Patients, 2005-2019

**DOI:** 10.1001/jamanetworkopen.2024.40414

**Published:** 2024-10-22

**Authors:** Thuy N. Thai, Joshua Brown, Stephan Schmidt, Judith Maro, Sonja A. Rasmussen, Almut G. Winterstein

**Affiliations:** 1 Department of Population Medicine, Harvard Pilgrim Health Care Institute and Harvard Medical School, Boston, Massachusetts; 2 Department of Pharmaceutical Outcomes and Policy, College of Pharmacy, University of Florida, Gainesville; 3 Center for Drug Evaluation and Safety (CoDES), University of Florida, Gainesville; 4 Department of Pharmaceutics, College of Pharmacy, University of Florida, Gainesville; 5 Johns Hopkins University School of Medicine, Baltimore, Maryland; 6 Department of Epidemiology, College of Medicine and College of Public Health and Health Professions, University of Florida, Gainesville

## Abstract

This cohort study investigates prescription antiemetic treatment patterns considering monotherapy, switching, and combination therapy during the first trimester of pregnancy and evaluates factors associated with ondansetron use.

## Introduction

According to the 2004 American College of Obstetricians and Gynecologists (ACOG) guideline, pyridoxine or a combination of doxylamine and pyridoxine is recommended as first-line therapy, whereas ondansetron should be reserved as a last-line therapy to control nausea and vomiting in pregnancy (NVP).^1^ The 2018 ACOG guideline recommends use of ondansetron as third-line therapy.^2^ Given the treatment guidelines’ updates, little is known regarding how antiemetics have been used. We aimed to address this evidence gap by describing prescription antiemetic treatment patterns considering monotherapy, switching, and combination therapy during the first trimester. We also evaluated factors associated with ondansetron use, the most commonly used prescription antiemetic during the study period.

## Methods

We used the Merative MarketScan Commercial Claims data from 2005 to 2019 to identify pregnant persons aged 12 to 55 years. Pregnancy outcomes and gestation duration were estimated with previously validated algorithms.^3,4^ The MarketScan data included approximately 20 to 40 million privately insured enrollees annually. We required pregnant persons to have continuous health plan enrollment for at least 90 days before estimated conception date to 30 days after pregnancy end (eFigure in Supplement 1). We classified prescription antiemetic treatment utilization based on pharmacy dispensing claims during the first trimester into monotherapy, switching, and combination therapy. Combination use was defined as a prescription fill for a second antiemetic during the active days’ supply of a different (first) antiemetic and a refill of the first antiemetic during the active days’ supply of the second.^5^ Use of multiple antiemetics that did not meet this combination definition was considered switching therapy. We conducted a stepwise selection with an entry and stay criterion of *P* < .10 to select factors potentially associated with using ondansetron vs alternatives and used generalized estimating equation models with a robust variance estimator to calculate risk ratios (Table). The analysis was completed on April 30, 2022. The University of Florida institutional review board considered the study exempt from human participants research review due to the use of deidentified data. This report followed the STROBE reporting guideline.

**Table.** Factors Associated With Ondansetron Use in Monotherapy, Switching, or Combination Therapy by Year of Conception

Description	Monotherapy				Switching therapy				Combination therapy			
	No./total No (%)	Adjusted risk ratio of ondansetron use (95% CI)^a^			No./total No (%)	Adjusted risk ratio of ondansetron use (95% CI)^a^			No./total No (%)	Adjusted risk ratio of ondansetron use (95% CI)^a^		
		2005-2006	2007-2012	2013-2019		2005-2006	2007-2012	2013-2019		2005-2006	2007-2012	2013-2019
Maternal age, y												
<20	6124/11 082 (55.3)	0.50 (0.43-0.59)^b^	0.80 (0.78-0.82)^b^	1.05 (1.03-1.08)	2416/2672 (90.4)	0.93 (0.85-1.02)	0.98 (0.97-1.00)	1.02 (1.00-1.04)	191/221 (86.4)	NS	NS	NS
20-24	28 828/48 745 (59.1)	0.72 (0.67-0.78)^b^	0.90 (0.89-0.91)	1.00 (0.99-1.01)	12 241/13 673 (89.5)	0.96 (0.92-1.00)	0.99 (0.99-1.00)	0.99 (0.98-1.01)	1317/1495 (88.1)	NS	NS	NS
25-29	68 125/108 566 (62.8)	1 [Reference]	1 [Reference]	1 [Reference]	27 301/30 044 (90.9)	1 [Reference]	1 [Reference]	1 [Reference]	3270/3674 (89)	NS	NS	NS
30-34	79 705/124 054 (64.3)	1.22 (1.17-1.28)^b^	1.06 (1.06-1.07)	0.97 (0.96-0.98)	28 519/31 161 (91.5)	1.04 (1.02-1.07)	1.01 (1.01-1.02)	1.00 (0.99-1.01)	3797/4278 (88.8)	NS	NS	NS
>34	43 995/69 667 (63.2)	1.26 (1.20-1.33)^b^	1.07 (1.06-1.07)	0.93 (0.92-0.95)	14 719/16 159 (91.1)	1.02 (0.99-1.05)	1.01 (1.00-1.01)	1.00 (0.99-1.01)	1992/2260 (88.1)	NS	NS	NS
Gestational age at index <9 wk	147 816/231 035 (64)	1.07 (1.03-1.11)	1.07 (1.06-1.07)	1.05 (1.04-1.06)	58 507/64 195 (91.1)	0.98 (0.96-1.00)	1.01 (1.01-1.02)	NS	7023/7949 (88.4)	NS	NS	0.97 (0.95-0.99)
Smoking	2215/3710 (59.7)	NS	0.95 (0.91-0.99)	1.07 (1.03-1.11)	1004/1117 (89.9)	0.81 (0.60-1.11)	NS	NS	139/154 (90.3)	NS	NS	NS
Alcohol/substance related disorders	1583/2876 (55.0)	NS	NS	0.85 (0.81-0.89)^b^	769/883 (87.1)	NS	NS	NS	123/142 (86.6)	NS	NS	NS
Hospitalization	1375/2283 (60.2)	NS	0.94 (0.90-0.98)	1.13 (1.08-1.19)	490/541 (90.6)	NS	NS	NS	51/54 (94.4)	NS	NS	NS
Emergency department visit	12 480/20 655 (60.4)	NS	0.96 (0.94-0.97)	NS	5643/6354 (88.8)	NS	0.99 (0.98-1.00)	0.97 (0.96-0.99)	658/749 (87.9)	NS	NS	NS
No. of physician office visits												
0	88 486/141 532 (62.5)	1 [Reference]	1 [Reference]	1 [Reference]	31 520/34 833 (90.5)	NS	1 [Reference]	NS	3837/4346 (88.3)	NS	NS	NS
1	55 626/88 859 (62.6)	1.05 (1.00-1.10)	1.02 (1.01-1.02)	0.98 (0.97-0.99)	20 928/22 990 (91)	NS	1.01 (1.00-1.02)	NS	2543/2838 (89.6)	NS	NS	NS
2	31 245/49 880 (62.6)	1.06 (1.00-1.13)	1.03 (1.02-1.04)	0.97 (0.96-0.99)	12 195/13 411 (90.9)	NA	1.01 (1.00-1.01)	NS	1512/1712 (88.3)	NS	NS	NS
>2	51 420/81 827 (62.8)	1.14 (1.09-1.20)	1.05 (1.04-1.06)	0.96 (0.94-0.97)	20 553/22 477 (91.4)	NS	1.01 (1.01-1.02)	NS	2675/3032 (88.2)	NS	NS	NS
No. of distinct filled generic ingredients												
0	102 317/162 151 (63.1)	1 [Reference]	1 [Reference]	NS	35 756/39 427 (90.7)	NS	NS	NS	4396/4975 (88.4)	NS	NS	NS
1	49 228/78 065 (63.1)			NS	17 960/19 725 (91.1)	NS	NS	NS	2291/2579 (88.8)	NS	NS	NS
>1	75 232/121 892 (61.7)	0.96 (0.91-1)	0.97 (0.96-0.98)	NS	31 480/34 555 (91.1)	NS	NS	NS	3880/4374 (88.7)	NS	NS	NS
Cervical cancer screening	30 885/48 356 (63.9)	NS	0.99 (0.98-1.00)	1.04 (1.02-1.05)	11 507/12 564 (91.6)	NS	0.99 (0.99-1.00)	NS	1409/1571 (89.7)	NS	NS	NS
Clinical characteristics associated with NVP												
Multiple gestation	2213/3419 (64.7)	NS	1.08 (1.05-1.11)	0.94 (0.90-0.99)	890/984 (90.5)	NS	1.04 (1.03-1.05)	0.95 (0.91-0.99)	189/220 (85.9)	NS	NS	NS
Overweight or obesity	5510/9939 (55.4)	0.62 (0.42-0.90)^b^	NS	0.88 (0.86-0.90)^b^	2422/2824 (85.8)	NS	0.98 (0.96-1.00)	0.96 (0.94-0.98)	316/366 (86.3)	NS	NS	NS
Preexisting diabetes	2922/4833 (60.5)	NS	0.96 (0.93-0.99)	NS	1126/1245 (90.4)	NS	NS	NS	161/189 (85.2)	NS	NS	NS
Asthma	4621/7241 (63.8)	NS	1.03 (1.01-1.06)	1.03 (1.00-1.06)	1885/2097 (89.9)	NS	NS	NS	275/324 (84.9)	NS	NS	NS
Gestational anemia	265/483 (54.9)	NS	0.92 (0.83-1.02)	0.87 (0.77-0.98)	142/162 (87.7)	NS	0.95 (0.88-1.03)	NS	31/38 (81.6)	NS	NS	NS
Depression	8808/14 034 (62.8)	NS	NS	1.05 (1.03-1.07)	3879/4269 (90.9)	NS	NS	1.02 (1.00-1.03)	583/667 (87.4)	NS	NS	NS
Anxiety disorder	10 113/16 495 (61.3)	NS	1.03 (1.02-1.05)	0.96 (0.94-0.98)	4475/4962 (90.2)	NS	NS	NS	729/825 (88.4)	NS	NS	NS
Attention-deficit and other childhood disorders^c^	2480/3940 (62.9)	NS	1.08 (1.05-1.12)	1.03 (1.00-1.07)	1061/1176 (90.2)	NS	1.03 (1.01-1.04)	NS	115/134 (85.8)	NS	NS	NS
Bipolar disorder	1624/2599 (62.5)	NS	NS	1.06 (1.01-1.11)	716/784 (91.3)	NS	NS	NS	87/96 (90.6)	NS	NS	NS
Other mental health disorders	1415/3080 (45.9)	NS	1.1 (1.01-1.19)	0.76 (0.73-0.79)^b^	776/940 (82.6)	NS	NS	0.94 (0.91-0.97)	151/172 (87.8)	NS	NS	NS
Differential diagnoses of NVP	27 501/44 464 (61.9)	0.93 (0.88-0.99)	0.98 (0.97-0.99)	1.01 (1.00-1.03)	11 947/13 181 (90.6)	NS	NS	NS	1690/1928 (87.7)	NS	NS	NS
Nausea and vomiting severity indicators and associated complications												
Nausea and vomiting diagnosis	22 991/36 517 (63)	0.86 (0.78-0.94)	1.05 (1.03-1.06)	0.98 (0.97-1.00)	13 953/15 545 (89.8)	NS	1.01 (1.00-1.02)	NS	3098/3542 (87.5)	0.84 (0.75-0.95)	NS	NS
Mild hyperemesis gravidarum	12 161/17 569 (69.2)	1.21.00 (1.12-1.31)^b^	1.09 (1.08-1.11)	1.1 (1.08-1.12)	10 076/11 035 (91.3)	1.06 (1.04-1.09)	1.02 (1.01-1.02)	NS	3458/3897 (88.7)	NS	NS	NS
Hyperemesis gravidarum with metabolic disturbance	2676/3765 (71.1)	1.25 (1.10-1.41)^b^	1.06 (1.04-1.09)	1.06 (1.02-1.11)	2995/3254 (92.0)	NS	1.01 (1.00-1.02)	NS	1476/1663 (88.8)	1.14 (1.05-1.23)	NS	NS
Electrolyte or laboratory abnormalities	1425/2278 (62.6)	NS	NS	NS	848/955 (88.8)	NS	NS	NS	284/320 (88.8)	NS	NS	NS
Dehydration	3222/4514 (71.4)	1.30 (1.12-1.51)^b^	1.06 (1.03-1.08)	1.13 (1.09-1.17)	2806/3063 (91.6)	1.06 (1.01-1.10)	NS	NS	1080/1201 (89.9)	NS	1.02 (1.00-1.04)	NS
Hospitalization with NVP related diagnosis	414/649 (63.8)	NS	0.94 (0.87-1.00)	NS	629/673 (93.5)	NS	NS	1.06 (1.02-1.11)	479/545 (87.9)	NS	0.94 (0.90-0.98)	NS
Clinical characteristics associated with QT prolongation												
Hypertension	3644/6106 (59.7)	NS	0.92 (0.89-0.94)	1.04 (1.00-1.07)	1401/1578 (88.8)	NS	NS	NS	173/200 (86.5)	NS	NS	NS
Other cardiovascular conditions	1823/2821 (64.6)	NS	NS	1.12 (1.07-1.17)	700/765 (91.5)	NS	NS	NS	105/117 (89.7)	NS	NS	NS
Diarrhea	2170/3319 (65.4)	NS	NS	1.06 (1.02-1.10)	1117/1228 (91)	NS	NS	NS	216/237 (91.1)	NS	NS	NS
Autoimmune disease	2100/3332 (63)	NS	NS	NS	769/851 (90.4)	NS	NS	NS	112/134 (83.6)	NS	NS	0.89 (0.78-1.01)
Epilepsy	744/1196 (62.2)	1.11 (0.7-1.77)	NS	NS	291/320 (90.9)	NS	NS	NS	35/40 (87.5)	NS	NS	NS
Previous cesarean	1354/2133 (63.5)	NS	0.93 (0.89-0.97)	1.13 (1.07-1.18)	493/534 (92.3)	NS	0.97 (0.93-1.00)	1.09 (1.05-1.13)	85/92 (92.4)	NS	NS	NS
Medication use												
Drugs with known QT prolongation before conception	34 158/54 470 (62.7)	1.05 (0.99-1.11)	NS	NS	14 579/15 907 (91.7)	NS	NS	1.01 (1.00-1.02)	1803/2045 (88.2)	NS	NS	NS
Drugs with known QT prolongation at index	7327/12 354 (59.3)	0.83 (0.73-0.94)	0.94 (0.92-0.96)	NS	2997/3298 (90.9)	NS	NS	NS	370/427 (86.7)	NS	NS	NS
Drugs with possible risk of QT prolongation before conception	22 122/36 153 (61.2)	0.92 (0.86-0.99)	0.97 (0.96-0.98)	1.05 (1.03-1.06)	9687/10 596 (91.4)	NS	NS	NS	1182/1323 (89.3)	NS	NS	NS
Drugs with possible risk of QT prolongation at index	6288/11 303 (55.6)	0.62 (0.53-0.71)^b^	0.8 (0.79-0.82)^b^	1.07 (1.04-1.09)	2555/2793 (91.5)	0.92 (0.85-1.01)	0.99 (0.97-1.00)	1.04 (1.02-1.06)	279/313 (89.1)	NS	NS	NS
Any selected Inhibitors of CYP1A2	1957/3244 (60.3)	NS	1.02 (0.99-1.06)	NS	734/798 (92.0)	NS	1.02 (1.00-1.04)	NS	91/102 (89.2)	NS	NS	NS
Any selected inhibitors of CYP2D6	11 348/18 348 (61.9)	NS	1.02 (1.01-1.04)	NS	5260/5791 (90.8)	NS	NS	NS	806/901 (89.5)	NS	NS	NS
Any selected inhibitors of CYP3A4	2722/4782 (56.9)	NS	0.88 (0.85-0.92)	1.04 (1.01-1.08)	1256/1405 (89.4)	0.91 (0.81.00-1.03)	0.97 (0.95-1.00)	NS	235/269 (87.4)	NS	NS	NS
No. of antiemetic before index												
0	NA	NA	NA	NA	NA	NA	NA	NA	2943/3360 (87.6)	1 [Reference]	1 [Reference]	1 [Reference]
1	NA	NA	NA	NA	NA	NA	NA	NA	5586/6149 (90.8)	NS	0.93 (0.82-1.06)	0.84 (0.80-0.88)^b^
>1	NA	NA	NA	NA	NA	NA	NA	NA	2038/2419 (84.3)	NS	0.90 (0.79-1.02)	0.82 (0.78-0.87)^b^
First antiemetic												
Ondansetron	NA	NA	NA	NA	NA	NA	NA	NA	5286/5570 (94.9)	NS	1 [Reference]	1 [Reference]
Promethazine	NA	NA	NA	NA	NA	NA	NA	NA	1189/1464 (81.2)	NS	0.94 (0.91-0.97)	0.78 (0.74-0.82)^b^
Metoclopramide	NA	NA	NA	NA	NA	NA	NA	NA	489/609 (80.3)	NS	0.96 (0.92-1.01)	0.78 (0.72-0.83)^b^
Others	NA	NA	NA	NA	NA	NA	NA	NA	3603/4285 (84.1)	NS	0.89 (0.78-1.02)	0.75 (0.71-0.78)^b^
Clinician type												
Obstetrics and Genecology	56 923/92 168 (61.8)	1 [Reference]	1 [Reference]	1 [Reference]	21 174/23 083 (91.7)	1 [Reference]	1 [Reference]	1 [Reference]	2597/2931 (88.6)	NS	NS	1 [Reference]
Family practice	8342/14 435 (57.8)	0.48 (0.41-0.55)^b^	0.80 (0.78-0.81)^b^	1.23 (1.21-1.26)^b^	2869/3166 (90.6)	0.90 (0.82-0.98)	0.96 (0.95-0.98)	1.04 (1.02-1.06)	254/287 (88.5)	NS	NS	1.01 (0.95-1.07)
Emergency medicine	4596/6483 (70.9)	0.62 (0.47-0.82)^b^	1.06 (1.04-1.08)	1.28 (1.25-1.31)^b^	2317/2650 (87.4)	0.79 (0.68-0.92)	0.98 (0.96-0.99)	0.96 (0.94-0.98)	349/410 (85.1)	NS	NS	0.95 (0.89-1.01)
Other types	94 038/148 138 (63.5)	0.89 (0.85-0.93)^b^	0.99 (0.98-1.00)	1.11 (1.10-1.12)^b^	36 616/40 617 (90.2)	0.97 (0.95-1.00)	0.98 (0.98-0.99)	0.99 (0.98-1.00)	5108/5768 (88.6)	NS	NS	1.00 (0.98-1.03)
Unknown	62 878/100 879 (62.3)	0.96 (0.91-1.00)	0.99 (0.98-0.99)	1.06 (1.05-1.07)	22 220/24 194 (91.8)	0.99 (0.96-1.01)	1.00 (0.99-1.00)	1.01 (1.00-1.02)	2259/2532 (89.2)	NS	NS	1.00 (0.97-1.04)

Abbreviations: NS, not selected; NVP, nausea and vomiting during pregnancy.

^a^ NS cells indicate that variable was not selected into the final model.

^b^ The cell indicates that the evaluated variable was associated with at least 10% decreased or increased utilization of ondansetron.

^c^ Attention-deficit and other childhood disorders include attention-deficit, conduct, and disruptive behavior disorders, developmental disorders, and disorders usually diagnosed in infancy, childhood, or adolescence.

## Results

Among 3 303 463 included pregnancies, prescription antiemetics were used by 471 206 (14.3%); and among those receiving prescription antiemetics, maternal age distribution was 127 973 [30.7%] 25 to 29 years, 141 737 [34.0%] 30 to 34 years, 78 279 [18.8%] greater than 34 years. The prevalence of prescription monotherapy, switching, and combination therapy was 1096, 314, and 38 per 10 000 pregnancies. The most common patterns included use of ondansetron for monotherapy, promethazine and switching to ondansetron for switching, and promethazine-ondansetron for combination (Figure). In 2005 to 2006, ondansetron monotherapy was associated with greater NPV severity. From 2007, ondansetron monotherapy was more likely associated with clinician types (clinicians other than obstetrician-gynecologists) but not with maternal NVP severity or other clinical characteristics (Table). No factors were found for switching, whereas antiemetic treatment history was found to be associated with ondansetron combination.

**Figure.**
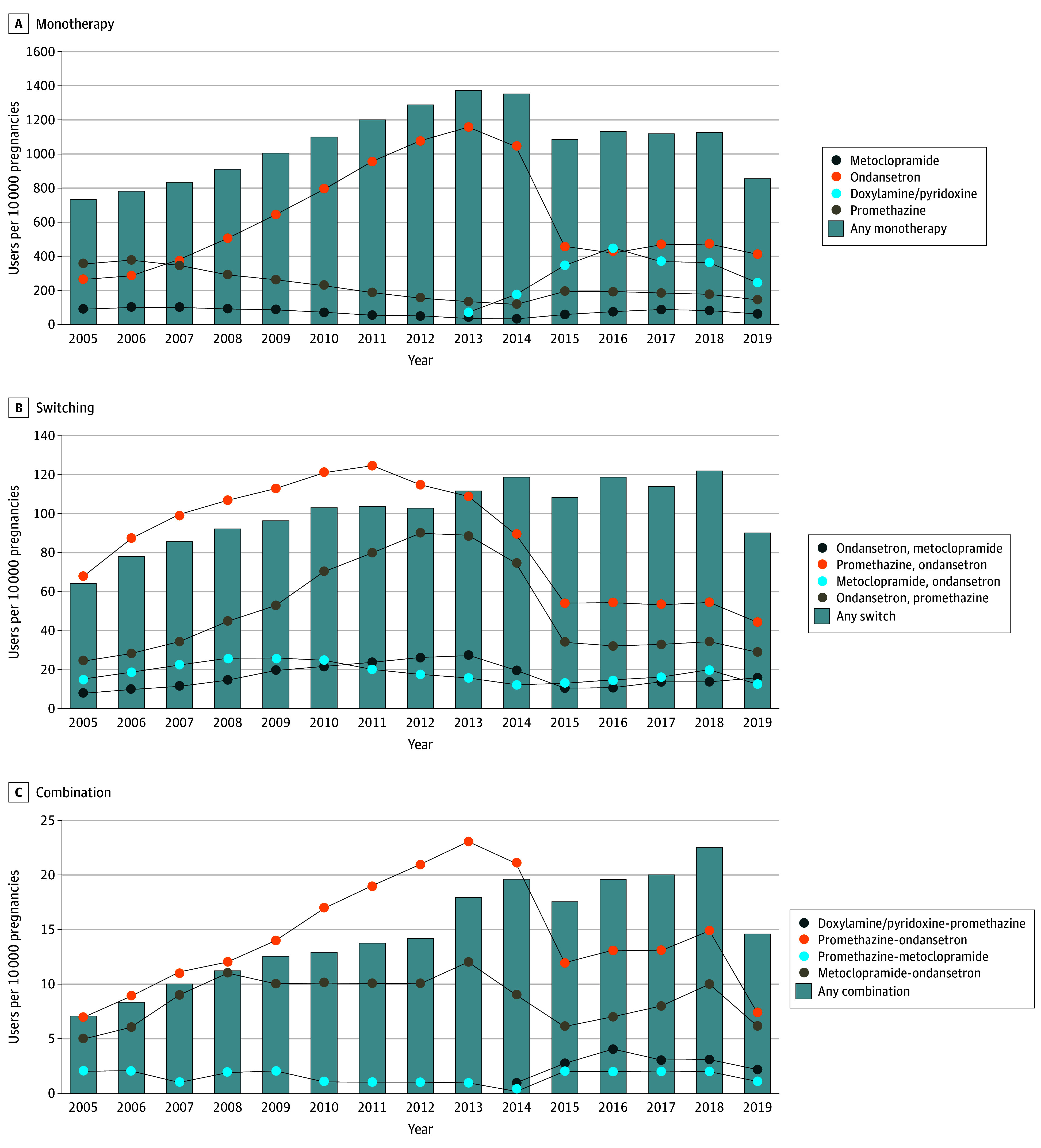
Annual Prevalence of Prescription Antiemetic Monotherapy, Switching, and Combination Therapies During the First Trimester in MarketScan Databases 2005-2019

## Discussion

Our study provides an understanding of prescription antiemetic utilization among US privately insured pregnant patients. Ondansetron was the most common prescription antiemetic and was dominantly used in monotherapy, switching, and combination therapy. The rapidly increasing utilization of ondansetron since 2006 aligned with the approval of generic versions with lower costs in conjunction with relaxed authorization policies.^6^ Starting in 2007, we found that ondansetron use was associated with clinician types rather than maternal characteristics including NVP severity, although recommendations place it as third-line therapy after other options have failed.

A limitation of our study was that we could not capture over-the-counter (OTC) antiemetics in our claims data. It is possible that patients initiated OTC monotherapy or combinations (eg, pyridoxine + doxylamine) before using ondansetron, which was observed as the first-line prescription antiemetic in our data. Moreover, claims-based measurement could misclassify NVP severity. Additionally, our study included a national sample of the privately insured pregnant population and due to average short enrollment spans in this population, imposing maternal enrollment requirements resulted in a loss of about half the initial sample size. Thus, our findings cannot be generalized to the entire US population, particularly the publicly insured population.

## Notes

Supplement 1. **eFigure.** Design diagram for evaluating determinants associated with ondansetron use

Supplement 2.
Data Sharing Statement
