# Supplementary material for: Use of Prescription Antiemetics Among US Commercially Insured Pregnant Patients, 2005-2019
Source: JAMA Netw Open. 2024 Oct 22;7(10):e2440414. doi: 10.1001/jamanetworkopen.2024.40414 (PMC11581608; doi:10.1001/jamanetworkopen.2024.40414)
Supplement: Supplement 1. — eFigure. Design diagram for evaluating determinants associated with ondansetron use [file jamanetwopen-e2440414-s001.pdf]

Supplemental Online Content

Thai TN, Brown J, Schmidt S, Maro J, Rasmussen SA, Winterstein AG. Use of prescription antiemetics among US commercially insured pregnant patients, 2005-2019. *JAMA Netw Open.* 2024;7(10):e2440414.
doi:10.1001/jamanetworkopen.2024.40414

eFigure. Design Diagram for Evaluating Determinants Associated With Ondansetron Use

This supplemental material has been provided by the authors to give readers additional information about their work.

© 2024 Thai TN et al. *JAMA Network Open.*

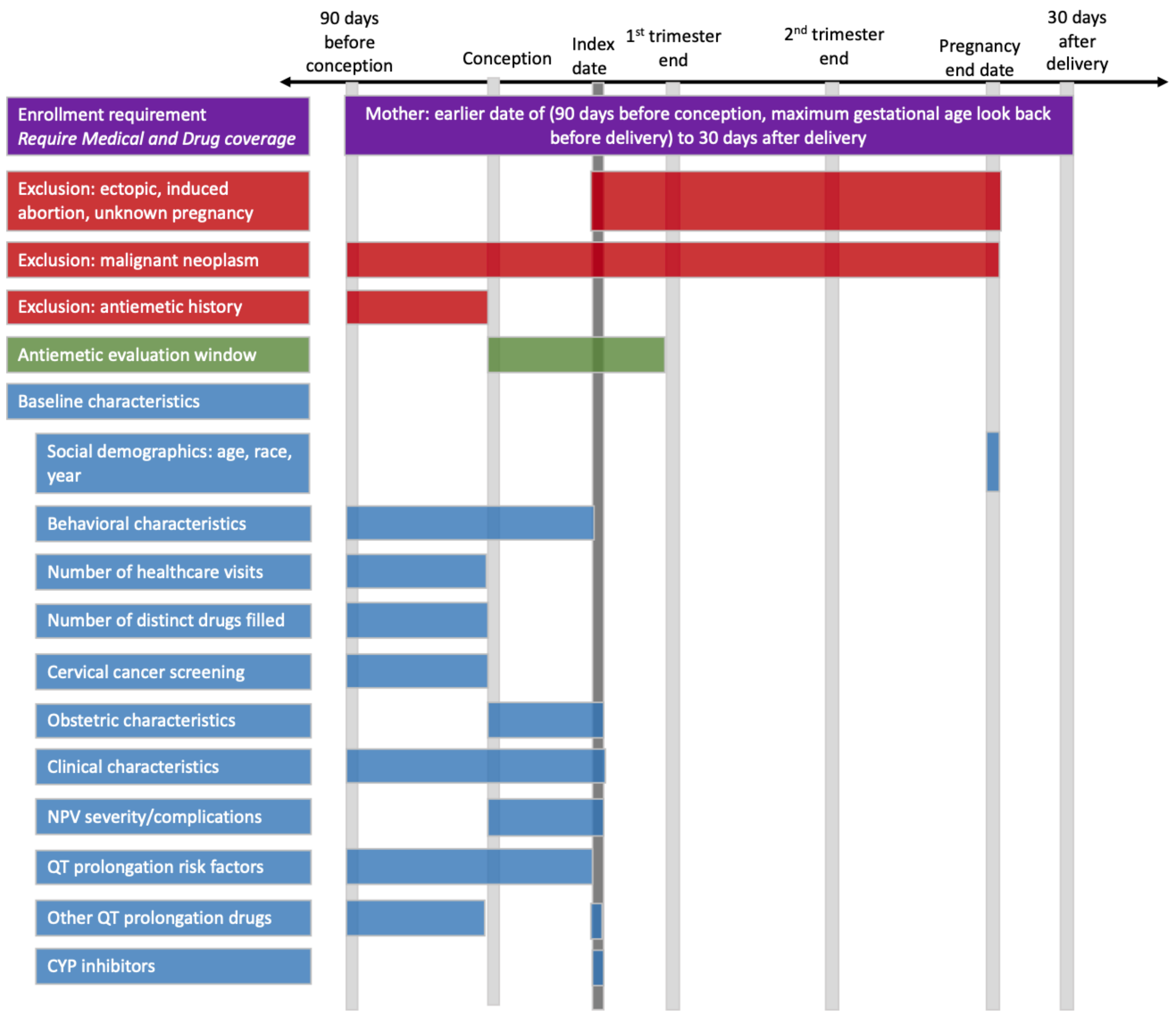

eFigure. Design diagram for evaluating determinants associated with ondansetron use

© 2024 Thai TN et al. *JAMA Network Open.*
