# Supplementary material for: Use of Prescription Antiemetics Among US Commercially Insured Pregnant Patients, 2005-2019
Source: JAMA Netw Open. 2024 Oct 22;7(10):e2440414. doi: 10.1001/jamanetworkopen.2024.40414 (PMC11581608; doi:10.1001/jamanetworkopen.2024.40414)
Supplement: Supplement 2. — Data Sharing Statement [file jamanetwopen-e2440414-s002.pdf]

Data Sharing Statement

Thai. Use of Prescription Antiemetics Among US Commercially Insured Pregnant Patients, 2005-2019. *JAMA Netw Open*. Published October 22, 2024.
doi:10.1001/jamanetworkopen.2024.40414

Data
Data available: No

Additional Information
Explanation for why data not available: Merative MarketScan data access is restricted by licensing agreements
